# RECTA: Regulon Identification Based on Comparative Genomics and Transcriptomics Analysis

**DOI:** 10.3390/genes9060278

**Published:** 2018-05-30

**Authors:** Xin Chen, Anjun Ma, Adam McDermaid, Hanyuan Zhang, Chao Liu, Huansheng Cao, Qin Ma

**Affiliations:** 1 Center for Applied Mathematics, Tianjin University, Tianjin 300072, China; chen_xin@tju.edu.cn; 2 Bioinformatics and Mathematical Biosciences Lab, Department of Agronomy, Horticulture and Plant Science, South Dakota State University, Brookings, SD 57006, USA; anjun.ma@sdstate.edu (A.Ma); Adam.McDermaid@sdstate.edu (A.Mc.); 3 Department of Mathematics and Statistics, South Dakota State University, Brookings, SD 57006, USA; 4 College of Computer Science and Engineering, University of Nebraska Lincoln, Lincoln, NE 68588, USA; unlzhhy@huskers.unl.edu; 5 Shandong Provincial Hospital affiliated to Shandong University, Jinan 250021, China; ibuliuchao@gmail.com; 6 Center for Fundamental and Applied Microbiomics, Biodesign Institute, Arizona State University, Tempe, AZ 85287, USA; hshcao@gmail.com

**Keywords:** RECTA, *Lactococcus lactis* MG1363, acid stress response, differentially expressed gene, gene co-expression, cis-regulatory motif finding, regulon, gene regulatory network

## Abstract

Regulons, which serve as co-regulated gene groups contributing to the transcriptional regulation of microbial genomes, have the potential to aid in understanding of underlying regulatory mechanisms. In this study, we designed a novel computational pipeline, regulon identification based on comparative genomics and transcriptomics analysis (RECTA), for regulon prediction related to the gene regulatory network under certain conditions. To demonstrate the effectiveness of this tool, we implemented RECTA on *Lactococcus lactis* MG1363 data to elucidate acid-response regulons. A total of 51 regulons were identified, 14 of which have computational-verified significance. Among these 14 regulons, five of them were computationally predicted to be connected with acid stress response. Validated by literature, 33 genes in *Lactococcus lactis* MG1363 were found to have orthologous genes which were associated with six regulons. An acid response related regulatory network was constructed, involving two trans-membrane proteins, eight regulons (*llrA*, *llrC*, *hllA*, *ccpA*, NHP6A, *rcfB*, regulons #8 and #39), nine functional modules, and 33 genes with orthologous genes known to be associated with acid stress. The predicted response pathways could serve as promising candidates for better acid tolerance engineering in *Lactococcus lactis*. Our RECTA pipeline provides an effective way to construct a reliable gene regulatory network through regulon elucidation, and has strong application power and can be effectively applied to other bacterial genomes where the elucidation of the transcriptional regulation network is needed.

## 1. Introduction

Genomic and transcriptomic analyses have been widely used for elucidating gene regulatory network (GRN) hierarchies and offering insight into the coordination of response capabilities in microorganisms [1,2,3,4]. One way to study the mechanism of transcriptional regulation in microbe genomics is regulon prediction. A regulon is a group of co-regulated operons, which contains single or multiple consecutive genes along the genome [5,6,7]. Genes in the same operon are controlled by the same promoter and are co-regulated by one or a set of transcriptional factors (TFs) [8]. The elucidation of regulons can improve the identification of transcriptional genes, and thus, reliably predict the gene transcription regulation networks [9].

There are three ways for regulon prediction: (i) predicting new operons for a known regulon [10,11]. This method combines motif profiling with a comparative genomic strategy to search for related regulon members and carries out systematical gene regulation study; (ii) Integrating cis-regulatory motif (motif for short) comparison and clustering to find significantly enriched motif candidates [12,13]. The candidate motifs are then assembled into regulons; (iii) Performing ab initio novel regulon inference using the de novo motif finding strategy [14]. This approach uses a phylogenetic footprinting technique which mostly relies on reference verification [15,16,17] and can perform a horizontal sequential comparison to predict regulons in target organisms by searching known functionally-related regulons or TFs from other relevant species. One algorithm for phylogenetic footprinting analysis called Motif Prediction by Phylogenetic footprinting (MP3) has been used for regulon prediction in *Escherichia coli* [17]. Phylogenetic footprinting was then integrated into the DMINDA webserver along with other algorithms, such as the Database of Prokaryotic Operons 2.0 (DOOR2) [7,18], Bottleneck Broken (BoBro) [19], and BoBro-based motif comparison (BBC) [13], to construct a complete pipeline for regulon prediction. In the latest research, a newly developed pipeline called Single-cell Regulatory Network Inference and Clustering (SCENIC) combines motif finding from co-expression gene modules (CEMs) with regulon prediction for single-cell clustering and analysis [20]. Such a method builds up a way of regulon application in single-cell and metagenomic research. Nevertheless, without a suitable regulon database, researchers need to build up the library first through operon identification, CEM analysis, motif prediction and comparison [21]. Here, we reported an integrated computational framework of regulon identification based on comparative genomics and transcriptomics analysis (RECTA) to elucidate the GRN responses in microbes under specific conditions. To better elucidate the methodology of RECTA, we built a regulatory network responding to the acid stress in *Lactococcus lactis* species.

*Lactococcus lactis* is one of the mesophilic Gram-positive lactic acid-producing bacteria. It has been widely applied in dairy fermentations, such as cheese and milk product [22]. Several studies have provided evidence of its essential roles in wrapping and delivering proteins or vaccinations for immune treatment, such as diabetes [23], malaria [24], tumors [25,26], and infections [27]. Holding the advantage of higher acid tolerance to protect vectors from resolving during delivery inside of the animal body, *L. lactis* has more potential and safety in oral drug development [28]. Moreover, it has been found that *L. lactis*, along with some *Lactobacillus*, *Bifidobacterium*, and other gut microbiota, were associated with obesity [29]. Such studies lead to the possibility and availability of *L. lactis* in metagenomic studies to investigate the effect of microbial interaction between *L. lactis* and other species in the human body. It is now well established that *Lactococcus* have evolved stress-sensing systems, which enable them to tolerate harsh environmental conditions [1,30,31]. 

Among the harsh environmental conditions that microorganisms confront, acid stress is known to change the level of the alarmones (guanosine tetraphosphate and guanosine pentaphosphate), collectively referred to as (p)ppGpp [32] and leads to a stringent response to cellular regulation [33]. The reason that bacteria maintain the protection mechanism against acid stress is to withstand the deleterious effects caused by the harmful high level of protons in the exposed environment. Many mechanisms or genes related to the acid stress response (ASR) have been identified. Proton-pumping activity, the direct regulator to acid stress response, controls the intracellular pH level by pumping extra protons out of the cell [34,35], and the increase of alkaline compound levels also counters the acidification found in *Streptococcus* [36]. Acid damage repair of cells by chaperones or proteases, such as GroES, GroEL, GrpE, HrcA, DnaK, DnaJ, Clp [37,38], hdeA/B and Hsp31 in *E. coli* [39,40], the arginine deiminase (ADI) system [41,42,43,44] and glutamate decarboxylases (GAD) pathways, and so on [45,46,47], have been proven to be associated with the acid response. Additionally, transcriptional regulators, σ factors, and two-component signal transduction system (TCSTs) have also been demonstrated to be responsible for ASR by modifying gene expression [48]. These genes or pathways suggest low pH has widespread adverse effects on cell functions and inflicts response at genomic, metabolic, and macromolecular levels. To better understand the mechanism that controls the acid tolerance and response to the acid stress in *L. lactis*, we considered MG1363, a strain extensively studied for acid resistance, to carry out computational analyses [1,49,50,51]. Nevertheless, to adequately describe the transcriptional state and gene regulation responsible for ASR in *L. lactis*, a GRN integrating all individual pathways is needed.

**Figure 1:**
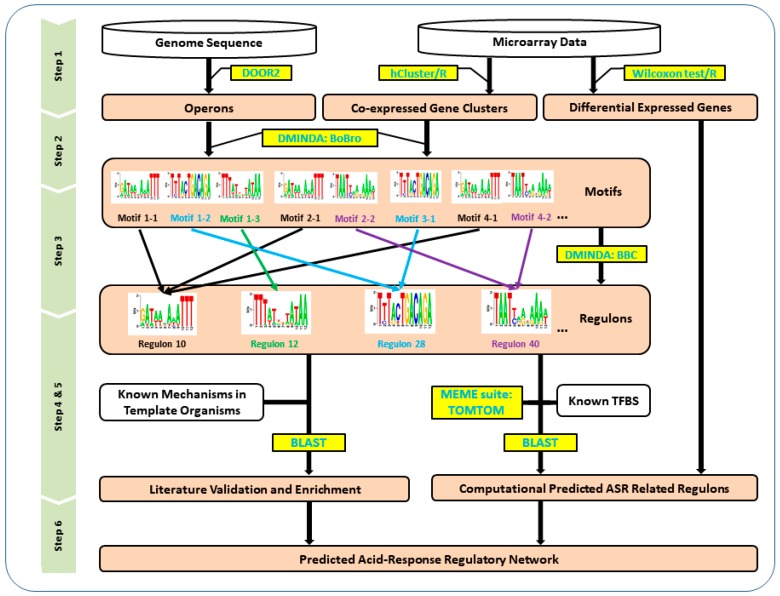
The flowchart of constructing the global acid stress response (ASR) transcriptional network in MG1363. Step 1: microarray data was used to generate co-expressed gene clusters and differentially expressed genes (DEGs), and the MG1363 genome sequence was used to find operons. Step 2: a motif finding progress was carried out to identify all statistically significant motifs in each of the co-expression gene modules (CEMs). Step 3: a regulon finding procedure was designed to identify all the possible regulon candidates encoded in the genome based on motif comparison and clustering. Step 4: the motifs of each of these regulons were compared to known transcription factor binding sites (TFBSs), and differential gene expression (DGE) analysis between low pH conditions and normal conditions was used to figure out the ASR-related regulons. Step 5: regulon validation based on literature information verified the significant putative regulons and expanded the results to some insufficiently significant regulons. Step 6: the ASR-related gene regulatory network (GRN) in MG1363 was predicted and described with eight regulons, nine functional modules, and 33 genes. The combination of the above information forms a genome-scale regulatory network constructed for ASR. Abbreviations: DOOR2, Database of Prokaryotic Operons 2.0; BBC, BoBro-based motif comparison; BLAST, basic local alignment search tool; BoBro, Bottleneck Broken.

The experiment was conducted by six steps and the general framework is showcased in Figure 1: (i) MG1363 co-expression gene modules (CEMs) and differentially expressed genes (DEG) were generated from microarray data by hcluster package [52] and Wilcoxon test [53] in R, respectively. MG1363 operons were predicted from the genome sequence using the DOOR2 webserver and assigned into each CEM; (ii) for each CEM, the 300 bp upstream to the promoter was extracted and the sequences were used to find motifs using DMINDA 2.0; (iii) the top five significant motifs in each CEM were reassembled by their similarity comparison and clustering to predict regulons; (iv) the motifs were compared to known transcription factor binding sites (TFBSs) in the MEME suite [54], and the TFs corresponding to these TFBSs were mapped to MG1363 using basic local alignment search tool (BLAST). Only regulons with DEGs and mapped TF were kept as ASR-related regulons; (v) experimentally identified ASR-related genes in other organisms were mapped to MG1363 using BLAST and allocated to corresponding regulons for further verification; and (vi) the relationship between regulons and functional gene modules was established to elucidate the overall ASR mechanism in MG1363.

As a result, 14 regulons are identified, literature verified or putative, to be connected to ASR. Eight regulons, related to nine functional modules and 33 associated genes, are considered as the essential elements in acid resistance in MG1363. This proposed computational pipeline and the above results significantly expand the current understanding of the ASR system, providing a new method to predict systematic regulatory networks based on regulon clustering.

## 2. Materials and Methods

### 2.1. Data Acquisition

The *L. lactic* MG1363 genome sequence was downloaded from NCBI (GenBank accession number: AM406671). The microarray dataset containing eight samples under different acid stress conditions for MG1363 was downloaded from the Gene Expression Omnibus (GEO) database (Series number: GSE47012). The data has been treated with LOWESS normalization by the provider. The details on cell culture preparation and data processing can be found in the previous study [1]. This dataset has all bacteria grown in basic conditions: a two-liter fermenter in chemically defined medium containing 1% (*w*/*v*) glucose at 30 °C. The control and treatment samples were grown at a pH of 6.5 and 5.1, respectively.

Several TFBS databases integrated in the MEME suite, including DPInteract (*E. coli*) [55], JASPAR [56], RegTransBase (prokaryotes) [57], Prodoric Release (prokaryotes) [58], and Yeastract (yeast) [59], were utilized for regulon filtering in known TF templates to find homologous TFs and corresponding genes in MG1363 using BLAST with default parameters. In the literature validation part, all ASR-related transporters and genes were collected from published articles, and their sequences were obtained from NCBI and UniProt databases.

### 2.2. Operon Identification

The genome-scale operons of MG1363 were identified by DOOR2. It is a one-stop operon-centered resource including operons, alternative transcriptional units, motifs, terminators, and conserved operons information across multiple species [18]. Operons were predicted by the back-end prediction algorithm with a prediction accuracy of 90–95% [60], based on the features of intergenic distance, neighborhood conservation, short DNA motifs, length ratio between gene pairs, and newly developed transcriptomic features trained from the strand-specific RNA sequencing (RNA-Seq) dataset [61,62].

### 2.3. Gene Differential Expression Analysis and Co-Expression Analysis 

Differentially expressed genes were identified based on the Wilcoxon signed-rank test [53] between the control and treatment, which was performed in R. The gene co-expression analysis was performed using a hierarchical clustering method (hcluster package in R) [52] to detect the CEMs under the acid stress in MG1363. 

### 2.4. Motif finding and Regulon Prediction

Genes from each CEM were first mapped to the identified operons to retrieve the basic transcription units. Next, 300 bps in the upstream of the translation starting sites for each operon were extracted, in which motif finding was carried out using the webserver DMINDA [63,64], with the whole genome sequence used as the control set. DMINDA is a dominant motif prediction tool, embraced five analytical algorithms to find, scan, and compare motifs [13,61,65], including a phylogenetic footprint framework to elucidate the mechanism of transcriptional regulation at a system level in prokaryotic genomes [9,17,19]. A motif length of 12 nucleotides was used as the representative length for regulon prediction [12,13]. The sequences were uploaded to the server and default parameters were used in the BBC program to conduct motif clustering to find the top five significant motifs (*p*-value < 0.05) in each cluster. The identified motifs were subjected to motif comparison and grouped into regulons using Kruskal’s algorithm with two similarity thresholds, T1 and T2, to give rise to the highly reliable and relatively reliable motif clusters, respectively, in the BBC program in DMINDA [13].

### 2.5. Regulon Validation Based on Transcription Factor BLAST and Differentially Expressed Gene Filtering

Each highly conserved motif was considered to contain the same TFBS among species. Therefore, a comparison study was performed using Tomtom with default parameters in the MEME Suite [54] between identified motif and public-domain TFBS databases, including DPInteract, JASPAR, RegTransBase, Prodoric Release and Yeastract, to find TFBSs and corresponding TFs with significant *p*-values in other prokaryotic species. Those TFs were then mapped to MG1363 using BLAST by default parameters to predict the connection between regulons and TFs in MG1363. On the other hand, since genes without differential expression were supposed not to react to pH changes, and thus, irrelevant to ASR, regulons without DEGs were not involved in the GRN, and thus, excluded from the following steps.

### 2.6. Regulon Validation Based on Known Acid Stress Response Proteins from the Literature

To validate the performance of the above computational pipeline for regulon prediction, a literature-based validation was performed. Thirty-six ASR-related proteins and genes in other organisms including *L. lactis*, *E. coli*, *Streptococcus*, and so on were first manually collected from literature, and their sequence was retrieved from the NCBI and UniProt databases. They were used to examine the existing known mechanisms in response to pH changes in MG1363 using the BLAST program by default parameters on NCBI. Such literature-based validation can either confirm the putative regulons when known ASR-related genes can be found in the significant regulons or expand our results to some insufficiently significant regulons, which indicate both false positive and true negative rate to evaluate the computational pipeline.

## 3. Results

### 3.1. Predicted Operons and Co-Expression Gene Module Generation

A total of 1565 operons with 2439 coding genes of MG1363 (dataset S1) were retrieved from the DOOR2 database. Through co-expression analysis, the 1565 operons were grouped into 124 co-expressed clusters by calculating the Euclidean distance using h = 0.05 × (MAX (distance)). Among these clusters, two large groupings contain more than 200 operons. Each of which was removed from the subsequent analyses as larger clusters may have higher chances to induce false positive operons which were connected with true operons by co-expression analysis. For the remaining 122 clusters covering 2122 genes, 26 (21%) contain no more than 10 operons; the smallest cluster had two operons, and most of the clusters (90%) contained between 10 and 50 operons (dataset S2 and Figure S1).

### 3.2. Predicted Regulons Based on Motif Finding and Clustering

Using BoBro in the DMINDA webserver, multiple motif sequences were identified from the 300 bps in the upstream of the translation start sites for each operon. Only the top five significant motifs (adjusted *p*-value < 0.001) were selected in each cluster, giving rise to a total of 610 (122 × 5) identified. The motif comparison-and-clustering analysis was then performed on the 610 motifs, and 51 motif clusters were identified, with a motif similarity 0.8 as a cutoff. Intuitively, the operons sharing highly similar motifs in each motif cluster are supposed to be regulated by the same TF and tend to be in the same regulon. Hence, these 51 motif clusters correspond to 51 regulons (dataset S3).

### 3.3. Computationally-Verified Regulon Based on Transcription Factor BLAST and Differential Gene Expression Analysis

Among the above 51 regulons, 14 were found containing motifs significantly (E-value < 0.05) matched to known TFBSs using TOMTOM in the MEME suite, representatively. The motif logos are shown in Figure S2, and more details can be found in dataset S4. The 14 TFBS-corresponding TFs were then mapped to MG1363 using BLAST to identify the real TFs/genes regulating each regulon. As a result, eight known TFs—spo0A, lhfB, GAL80, CovR, c4494, ihfA, CovR, and RHE_PF00288—were successfully mapped to MG1363 resulting in eight TFs with multiple hits. The gene *llrA* (llmg_0908) regulates regulons #12 and #37, *ccpA* (llmg_0775) regulated regulons #15 and #47, *hllA* (llmg_0496) regulates regulons #7 and #31 (Table 1). The genes *ccpA* [66,67], *llrA* [68], *llrC* [68], and *hllA* [69], were known to be ASR-related genes in *L. lactis*; the gene llmg_0271, without any related known TF, was found to be similar to template TF GAL80 in yeast, which has not been associated with any ASR regulation pathways yet. For all 14 significant regulons, regulons #3, #4, #20, #28, #40, and #44 are potential candidates as, currently, no related TFs in *L. lactis* have been found (Table 1).

Additionally, 86 down-regulated genes and 55 up-regulated genes (dataset S5), resulting from DGE analysis were integrated into the regulons. Regulons #10, #37, #44 and #47 were found to be lacking DEGs. Thus, gene llmg_0271, related to regulon #10, was not likely to respond to acid stress in MG1363 even though it has been successfully mapped to MG1363, and was then grouped into the potential candidate. On the contrary, *ccpA* and *llrA* were still retained due to their involvements in regulons #15 and #12 with DEGs, respectively.

By the end of the computational pipeline, we predicted that regulons #2, #7, #12, #15 and #31 were related to GRN in MG1363 (Figure S3). A hypergeometric algorithm was used to verify the possibility of the of DEG numbers in each regulon (dataset S6). Merging regulon #7 and #31 as one, we referred to their TF names (*ccpA*, *llrA*, *llrC*, and *hllA*) to represent the five regulons for convenience.

### 3.4. Verified Regulons Based on Literature Verification 

Altogether, 36 literature-supported ASR-related transporters were successfully mapped to MG1363 using blast with an E-value cutoff as 1e^−10^, which resulted in a total of 33 mapped genes. All the 36 transporters were categorized into nine modules based on their biological functions or regulated pathways, including L-lactate dehydrogenase (LDH), GAD, ADI, urea degradation, F1/F0ATPase, acid stress, protein repair and protease, envelope alterations, and DNA repair. The 33 mapped genes generate 22 operons and six regulons: *llrA*, *llrC*, *hllA*, NHP6A, regulon #8 and #39, which were subjected, one or more, to each functional module (Table 2). 

Regulons *llrA*, *llrC*, and *hllA* have already been computationally identified in Table 1 and supported again by literature verification results. The NHP6A gene, interestingly, has a homologous TF in humans and fungi but not in *L. lactis* [70,71], yet failed to map in MG1363. Here, we are using NHP6A to represent regulon #20, as their relationship has been predicted computationally in Table 1. Regulon #39 was identified to be regulated by *llrD*, one of the six two-component regulatory systems in MG1363 [68]. Regulons #8 (llmg_1803) and #39 (*llrD*) were not included in the 14 significant regulons in Table 1. For NHP6A, regulons #8 and #39 were enriched by literature validation as it expanded regulon results of the RECTA pipeline. Among the nine functional modules, *llrA* was found connected to five of them, and NHP6A related to three. On the other hand, the GAD and urea degradation functional modules failed to connect to any previous regulons. 

**Figure 2:**
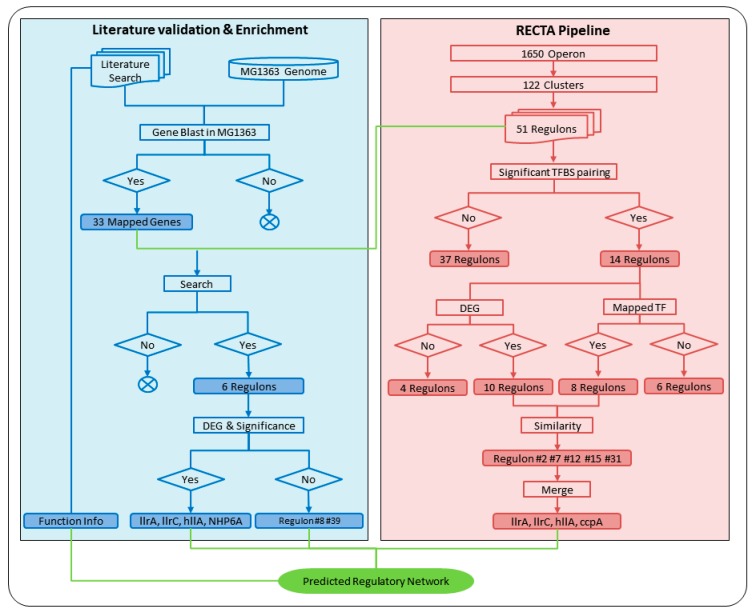
Regulon prediction using regulon identification based on comparative genomics and transcriptomics analysis (RECTA) pipeline (red) and validation and enrichment using literature information and gene blast (blue). All processes were shown in rectangles and results were highlighted with corresponding background colors. In the computational pipeline, 51 regulons with assigned motifs and operons were analyzed sequentially through significant TFBS pairing, DEG conformation, and TF BLAST. Only regulons contained DEGs (10) which had related mapped TF (8) were believed to be the final predicted ASR-related regulons (5). These five regulons were then merged into four, using the corresponding TFs to represent their names. In the literature validation process, known ASR-related transporters were first mapped to the MG1363 genome and resulted in 33 genes. Those genes were then searched in 51 regulons and determined six related regulons. All regulons resulting from both computational pipeline and literature validation were combined, along with the information of functional modules, to determine the GRN.

Compared to the regulon verification based on TF BLAST and DGE, the literature verification identified two more regulons (#8 and #39) that lay in the insignificant group, however, with no sign of *ccpA* regulon. Thus, such a result indicates a possible false positive rate of 1:5 and a true negative rate of 2:37 of our computational pipeline, indicating the reliability and feasibility of using RECTA to predict the ASR-related regulons. In Figure 2, we show the processes and results for both literature verification and the computational pipeline in detail. The final eight regulons predicted from both parts were then compared to construct a GRN response to acid stress, integrated with other information found in the literature.

### 3.5. A Model of Regulatory Network in Response to pH Change

**Figure 3:**
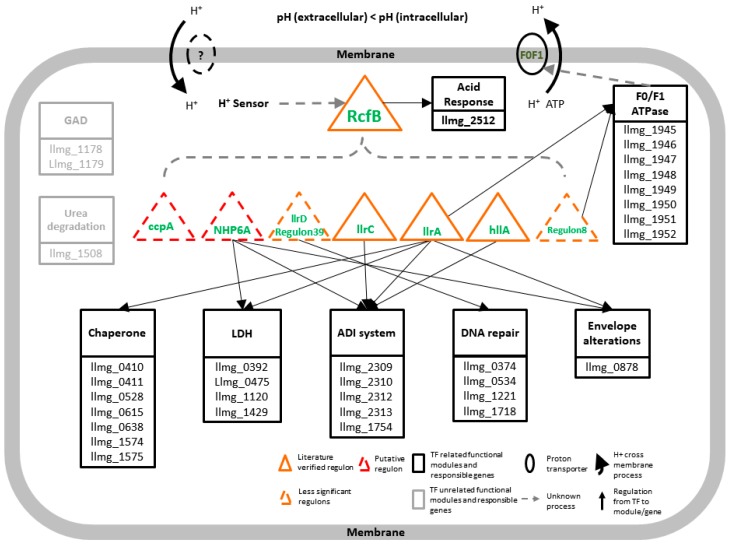
A working model of the transcriptional gene regulatory network in response to pH change in *L. lactis*. The mechanism is activated by the change of proton signal in a cell. Regulon *RcfB* is assumed to be the overall activator for the rest seven regulons and controls the ASR functional module solely. Three kinds of literature were verified; significant ASR-related regulons, *llrA*, *llrC*, and *hllA*, and two insufficiently significant regulons, *llrD* (regulon #39) and regulon #8 (llmg_1803) were predicted via our workflow but with results under a 0.8 motif similarity cutoff or a hit could not be found; one putative significant regulon NHP6A controls the seven functional modules which are experimentally verified in the close species MG1363. The other significant regulon *ccpA* failed to be confirmed by any literature-proved genes or transporters. Two extra functional modules, GAD, and urea degradation show no direct connection to all seven of the regulons. One or more homology genes are found in MG1363 for all the nine modules using BLAST. The solid arrows indicate regulation between regulons/TFs and functional modules/genes, and the dashed arrows indicate uncertain control processes. Additionally, two ovals indicate two trans-membrane proteins; one is confirmed as F0/F1ATPase and the other one, with the dashed line, whose related information we cannot find in the public-domain literature.

According to the results outlined above, we are presenting a working model of the transcriptional regulatory network for acid stress response in MG1363 (Figure 3). The network consists of two transmembrane proteins (dataset S7), eight regulons, nine functional modules, and 33 orthologous genes known for ASR in other bacteria that are also contributing in MG1363.

The network is subjected to respond to the change of intracellular proton level. The signal is captured by H^+^ sensor and regulons are initiated to be regulated. Although significance was not shown for *rcfB* in our computational results, it has been reported to recognize and regulate promoter P170 [72], P1, and P3 [73,74], which are activated by boxes A, C and D (ACiD-box) and essential to acid response [75]. With the ACiD box, operons like *groESL*, *lacticin 481* and *lacZ* have been proved to be regulated by *rcfB*, while *als*, *aldB*, etc., have not [75]. The homologous comparative study also predicted the existence of the ACiD box in *llrA* [68,76]. With such evidence, we separated *rcfB* from regulon #39 and predicted that *rcfB* is first triggered by H^+^ sensor and acts as the global initiator that controls the other seven regulons. It is reasonable that *rcfB*-related regulon #39 failed to show significant TF matching results after CEM treatment in the operon clustering step. The *rcfB* regulator worked as a trustworthy global factor; its differential expression should be less significant than regulons directly responding to acid stress, thus leading to the failure of being predicted by the RECTA pipeline. Nevertheless, the low number of microarray data sets (8) also limited the real performance to the ASR. However, the mechanism of how H^+^ sensor is activating and regulating the GRN and *rcfB* remains unclear. In the seven regulons, three—*llrA*, *llrC* and *hllA*—were verified through literature to be related to ASR; regulons #8 and #39 showed less significant in regulon prediction; NHP6A was considered as putative regulon due to its failure to map in MG1363; and *ccpA* was another putative regulon without literature support. 

The six downstream regulons (*llrA*, *llrC*, *hllA*, NHP6A, regulon #39, and regulon #8) other than *ccpA*, interact with each other to regulate six ASR-related functional modules, including the ADI system, DNA repair, LDH, protein repair, envelope alterations, and F0/F1 adenosine triphosphatase (F0/F1ATPase). The ADI pathway, which generates adenosine triphosphate (ATP) and protects cells from acid stress [44], is under the regulation of NHP6A, *llrC*, *llrA*, and *hllA*. Another important pathway is the LDH (EC 1.1.1.27) under the regulation of NHP6A and *llrA*, which converts pyruvate and H^+^ to lactate which is exported outside of cells [77]. Chaperons which take part in macromolecule protection and repairing are subjected to regulon *llrA*. Chaperons have functions that include providing protection to against environmental stress, helping protein folding, and repairing damaged proteins, and have been demonstrated to show clear linkage with acid stress in numerous Gram-positive bacteria [37,38,39,40]. The F0/F1ATPase, controlled by *llrA* and regulon #8, also plays an important role in maintaining normal cellular pH, which pumps H+ out of cells at the expense of ATP [34,35,78,79]. The GAD [45,46] and urea degradation [48] functional modules are missing reliable associations with the regulons in MG1363 while maintaining functions in ASR mechanism in other species.

## 4. Discussion and Conclusions

Implementation of the novel computational pipeline RECTA resulted in the construction of an eight-regulons enrolled ASR regulatory network. The framework provides a useful tool and will be a starting point toward a more systems-level understanding of the question [80]. The identified motifs and regulons suggest acid resistance is a coordinated response regarding regulons, although most of these have not been identified or experimentally verified. From the three well-identified regulons—*llrA*, *llrC*, and *hllA*—it appears the gene regulation is also complex, as these regulons also interact with other proteins and TFs. The F0/F1ATPase is directly involved in the concentration regulations of the intracellular proton. Other pathways are responsible for repairing the damage caused by acid stress, such as DNA repair, protein repair, and cell envelops alterations. However, there were also several reported ASR-related genes or transporters such as *htrA* in *Clostridium* spp. [81], CovS/CovR acid response regulator in *Streptococcus* [82], cyclopropane fatty acid (cfa) synthase for cell-membrane modification [83], and oxidative damage protectant genes like *sodA*, *nox-1* and *nox-2* [84] that failed to map to MG1363. Using more gene expression datasets for CEM and DGE analyses could be a way to strengthen the result of our computational pipeline, which might cover more significant regulons to construct a more solid and complete regulatory network.

Homology mapping at the genomic level showed very a long evolutionary distance between MG1363 and currently well-annotated model species. Hence, the functional analysis for MG1363 is limited, and it is hard to apply gene functional enrichment to verify our prediction results. With more expression datasets and experiments about protein–protein interactions, the ASR mechanism can be largely improved in *L. lactic* MG1363.

In summary, through the implementation of RECTA, we found that the ASR at the transcriptome level in MG1363 is an orchestrated complex network. Functional annotation shows these regulons are involved in many levels of biological processes, including but not limited to DNA expression, transcription, and metabolism. Our method builds a TF-regulons-GRN relationship so that the new ASR-related genes can be predicted. Besides, the low false positive and true negative rate indicate the RECTA pipeline as sensitive and reasonable. In fact, considering the high accuracy, we regarded *ccpA* as the putative regulon, though not connected to any related functional modules, while more robust methods are required. Such results expand current pathways to those that can corroborate cell structures—cell wall, cell membranes, and so on—and related functions. Our findings suggest that acid has profound adverse effects and inflicts a systems-level response. Such predicted response pathways can inform better resistance design. 

Looking forward to the acid tolerance advantage of *L. lactis*, which makes its prospective application in drug and vaccine delivery, the effects on anti-obesity research, and metagenomic studies, the ASR-related GRN in *L. lactis* shows an excellent research value. Fully understanding its theory may contribute to the development of *Lactococcus* therapy and can even expand to other close species by genetic modification. Furthermore, our computational pipeline provides an effective method to construct a reliable GRN based on regulon prediction, integrating CEMs, DGE analysis, motif finding, and comparative genomics study. It has a durable application power and can be effectively applied to other bacterial genomes, where the elucidation of the transcriptional regulation network is needed. 

In this study, we designed a computational framework, RECTA, for acid-response regulon elucidation. This tool integrates differential gene expression, co-expression analysis, cis-regulatory motif identification, and comparative genomics to predict and validate regulons associated with acid response. In demonstrating the efficacy of this tool, we analyzed *Lactococcus lactis* MG1363. This implementation resulted in the expanded understanding of the acid-response regulon network for this one strain of *L. lactis* and provides an applicable method for acid-response regulon elucidation of further species. Through utilization of the RECTA pipeline, researchers can readily evaluate acid-response mechanisms for numerous bacterial species, while simultaneously validating the results of their study. 

## Figures and Tables

**Table 1:** Altogether, 14 significant regulons that are verified and mapped to known transcription factors (TFs). According to analyses, operon numbers and DEG determination (yes or no), matched template TFs and mapped TFs were assigned for each significant regulon, respectively, and were aligned based on regulon ID number. Five regulons containing DEGs and having the corresponding TF at the same time were bolded, being computationally verified as the regulons responsible for acid stress in MG1363.

Regulon ID	No. of Operons	DEG	TF Template	TF (Gene) BLAST in MG1363
**Regulon #2**	82	Y	spo0A	*llrC* (*llmg_0414*)
Regulon #3	32	Y	FoxQ1	N/A
Regulon #4	20	Y	SPT2	N/A
**Regulon #7**	49	Y	lhfB	*hllA* (*llmg_0496*)
Regulon #10	5	N	GAL80	llmg_0271
**Regulon #12**	259	Y	CovR	*llrA* (*llmg_0908*)
**Regulon #15**	19	Y	c4494	*ccpA* (*llmg_0775*)
Regulon #20	79	Y	NHP6A	N/A
Regulon #28	5	Y	1Z916	N/A
**Regulon #31**	65	Y	ihfA	*hllA* (*llmg_0496*)
Regulon #37	10	N	CovR	*llrA* (*llmg_0908*)
Regulon #40	7	Y	Awh	N/A
Regulon #44	12	N	YBR182C	N/A
Regulon #47	5	N	RHE_PF00288	*ccpA* (*llmg_0775*)

Abbreviations: N, no; Y, yes; N/A, not found.

**Table 2:** Known ASR-related gene mapping from literature in response to pH change. Literature-supported ASR-related genes found in close species or other *Lactococcus lactis* strains. The template transporters and genes were first identified in published studies from the NCBI and UniProt databases. *Lactococcus lactis* Il1403 was used as the organism which is very close to MG1363 if template gene existed. Only 36 templates that successfully mapped to the MG1363 genome were listed, which resulted in 33 genes. All mapped genes and corresponding templated were organized by their regulated pathways which were further used as functional modules. Mapped genes were searched in 51 regulons to build the connections between functional modules and regulons.

Template Organisms			MG1363	
Organisms	Transporters	Functions/Pathways	Mapped Genes (Locus Tag)	Regulons
*Lactococcus lactis*	*ldh*	LDH	*ldh* (llmg_1120)	NHP6A, *llrA*
	*ldhB*		*ldhB* (llmg_0392, llmg_0475)	
	*ldhX*		*ldhX* (llmg_1429)	
*Lactococcus lactis*	*gadB*	GAD	*gadB* (llmg_1179)	N/A
	*gadC*		*gadC* (llmg_1178)	
*L actococcus lactis*	*arcA*	ADI pathway	*arcA* (llmg_2313)	NHP6A, *llrA*, *llrC*, *hllA*
	*arcB*		*arcB* (llmg_2312)	
	*arcC1*		*arcC1* (llmg_2310)	
	*arcC2*		*arcC2* (llmg_2309)	
	*argF*		*argF* (llmg_1754)	
Bacteria	*ureA/B/C* ^$^	Urea degradation	*pyrC* (llmg_1508)	N/A
*L actococcus lactis*	*atpEBFHAGDC* ^$$^	F0/F1ATPase	llmg_1952, llmg_1951, llmg_1950, llmg_1949, llmg_1948, llmg_1947, llmg_1946, llmg_1945	*llrA*, (Regulon8, llmg_1803) ^$$$^
*Lactococcus lactis*	*rcfB*	Acid response	rcfB (llmg_2512)	(Regulon39, *llrD*) ^$$$^
*Lactococcus lactis*, *Escherichia coli* K12	*dnak*	Chaperone, Protein repair and protease	*dnaK* (llmg_1574)	*llrA*
	*groEL*		*groEL2* (llmg_0411)	
	*groES*		*groES* (llmg_0410)	
	*grpE*		*grpE* (llmg_1575)	
	*clpE*		*clpE* (llmg_0528)	
	*clpC*		*clpC* (llmg_0615)	
	*clpP*		*clpP* (llmg_0638)	
*Lactococcus lactis*, *Bacillus subtilis*	*dltC*, *agK*, *SGP*, *ffh*	Envelope alterations	llmg_0878	NHP6A, *llrA*
*Lactococcus lactis*	*recA*, *uvr*, *smn*	DNA repair	llmg_0374, llmg_0534, llmg_1718 llmg_1221	(Regulon39, *llrD*) ^$$$^

^$^ Three subunits of urease enzymes coded by ureABC operon found preserved in multiple bacteria. ^$$^ Altogether eight genes. ^$$$^ The homolog prediction or motif research results with low homolog similarity but have meaningful biological relevance. Abbreviations: LDH, L-lactate dehydrogenase; GAD, glutamate decarboxylases; ADI, arginine deiminase; N/A, not found.

## Supplementary Materials

The following are available online at http://www.mdpi.com/2073-4425/9/6/278/s1. Figure S1: The distribution of operon numbers among 122 clusters, Figure S2: The motif logos extracted from 14 significant regulons, used for TFBS comparison. Figure S3: Heatmaps showing differential expression of the selected five regulons, Dataset S1: The operon prediction results for MG1363, Dataset S2: The gene clustering results for microarray GSE47012 containing operons. Dataset S3: The summarized motif prediction results for regulon prediction, Dataset S4: Computational validation results for predicted regulons, Dataset S5: 86 up-regulated genes and 55 down-regulated genes, Dataset S6: The possibility of the of DEG numbers in each regulon, Dataset S7: The prediction results for trans-membrane proteins.

## Abbreviations

ADI	Arginine deiminase
BBC	Bobro-based motif comparison
BoBro	Bottleneck broken
CEM	Co-expression (gene) module
DEG	Differentially expressed gene
DGE	Differential gene expression
*E. coli*	*Escherichia coli*
GAD	Glutamate decarboxylases
GRN	Gene regulatory network
LDH	Lactate dehydrogenase
*L. lactis*	*Lactococcus lactis*
MG1363	*Lactococcus lactis* MG1363
Motif	Cis-regulatory motif
RECTA	Regulon identification based on comparative genomics and transcriptomics analysis
TF	Transcription factors
TFBS	Transcriptional factor binding site
