# Supplementary material for: RECTA: Regulon Identification Based on Comparative Genomics and Transcriptomics Analysis
Source: Genes (Basel). 2018 May 30;9(6):278. doi: 10.3390/genes9060278 (PMC6027394; doi:10.3390/genes9060278)

Gene expression in Regulon #2 (*llrC*)

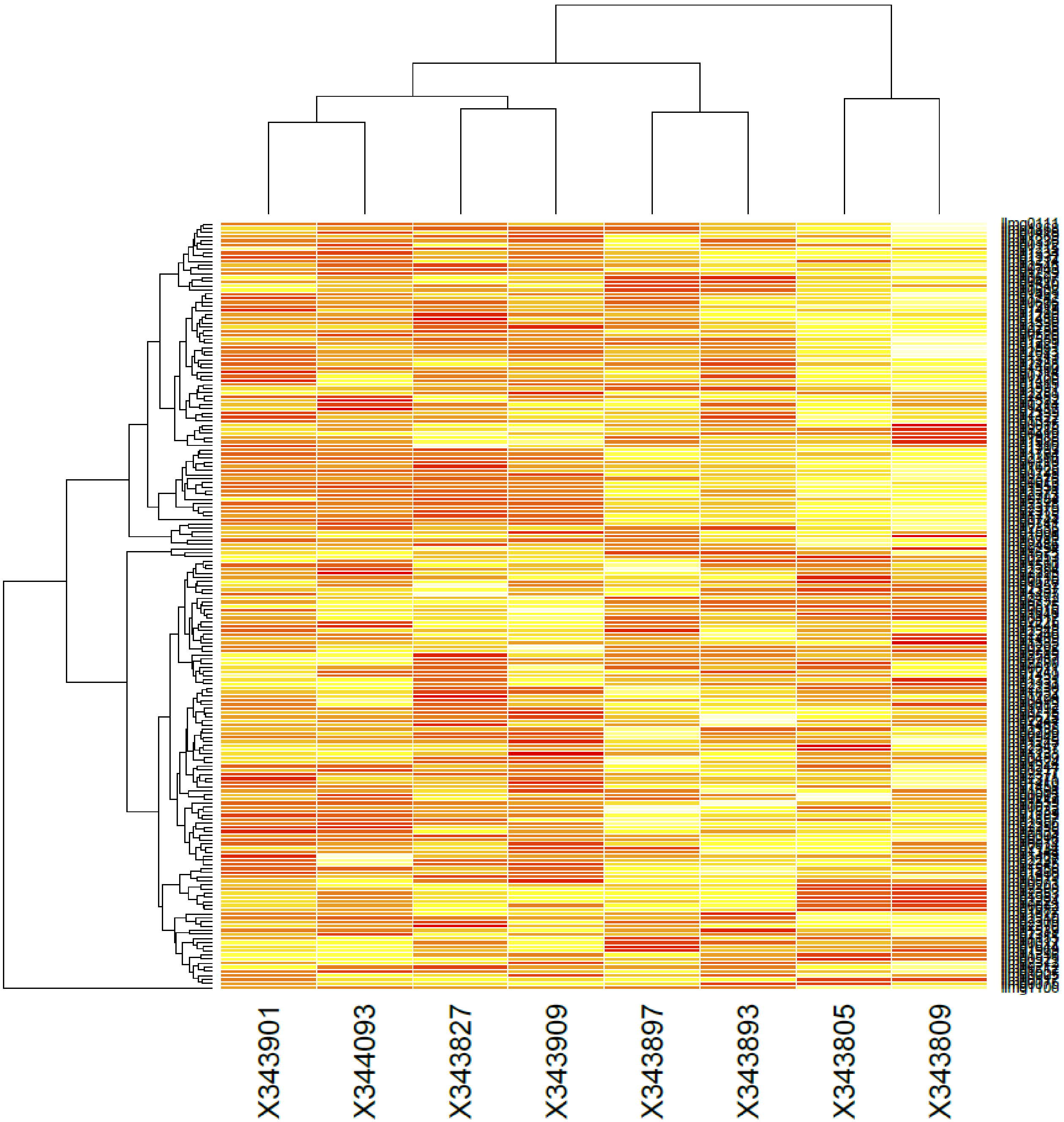

Gene expression in Regulon #7 (*hllA*)

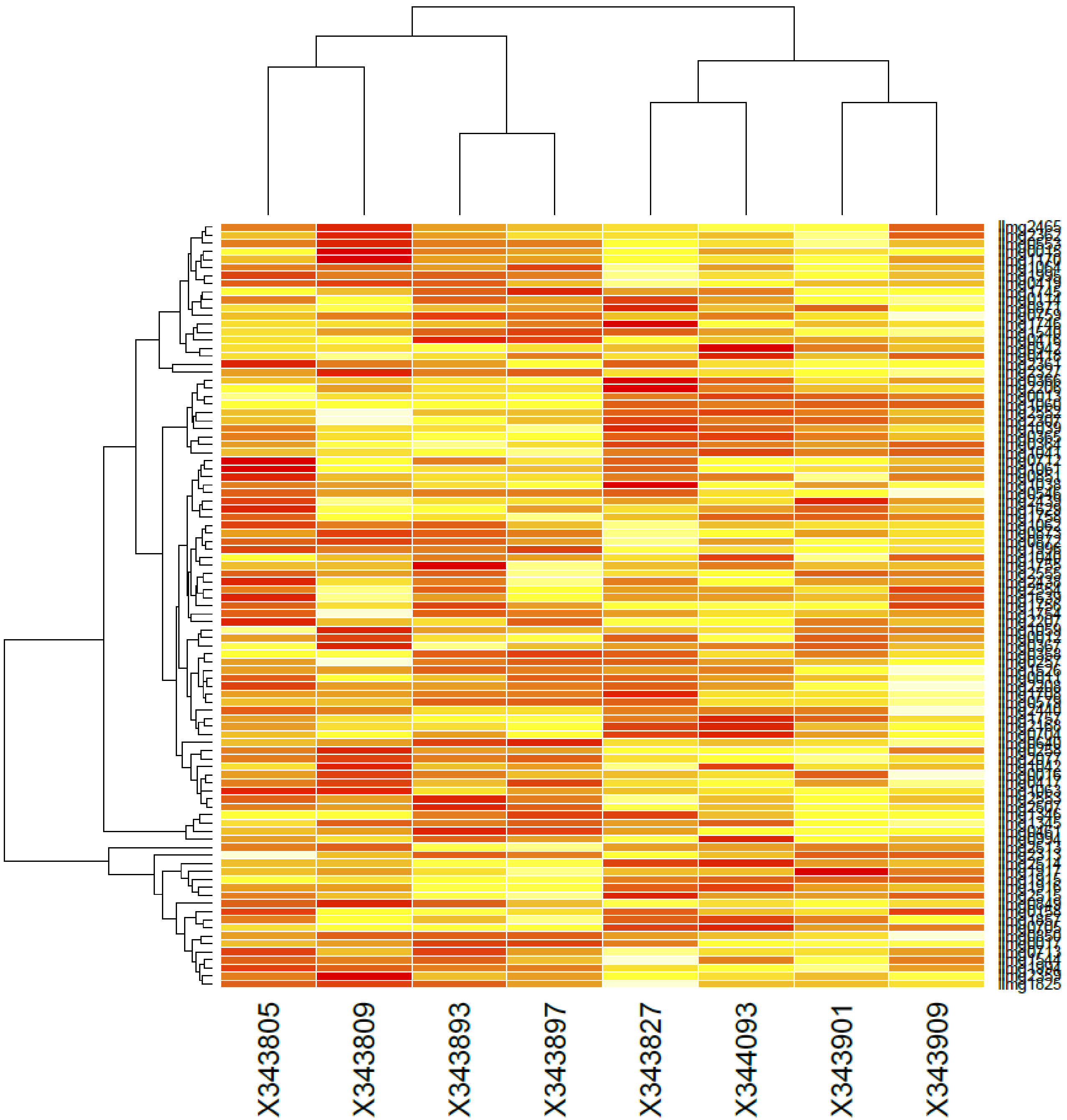

Gene expression in Regulon #12 (*llrA*)

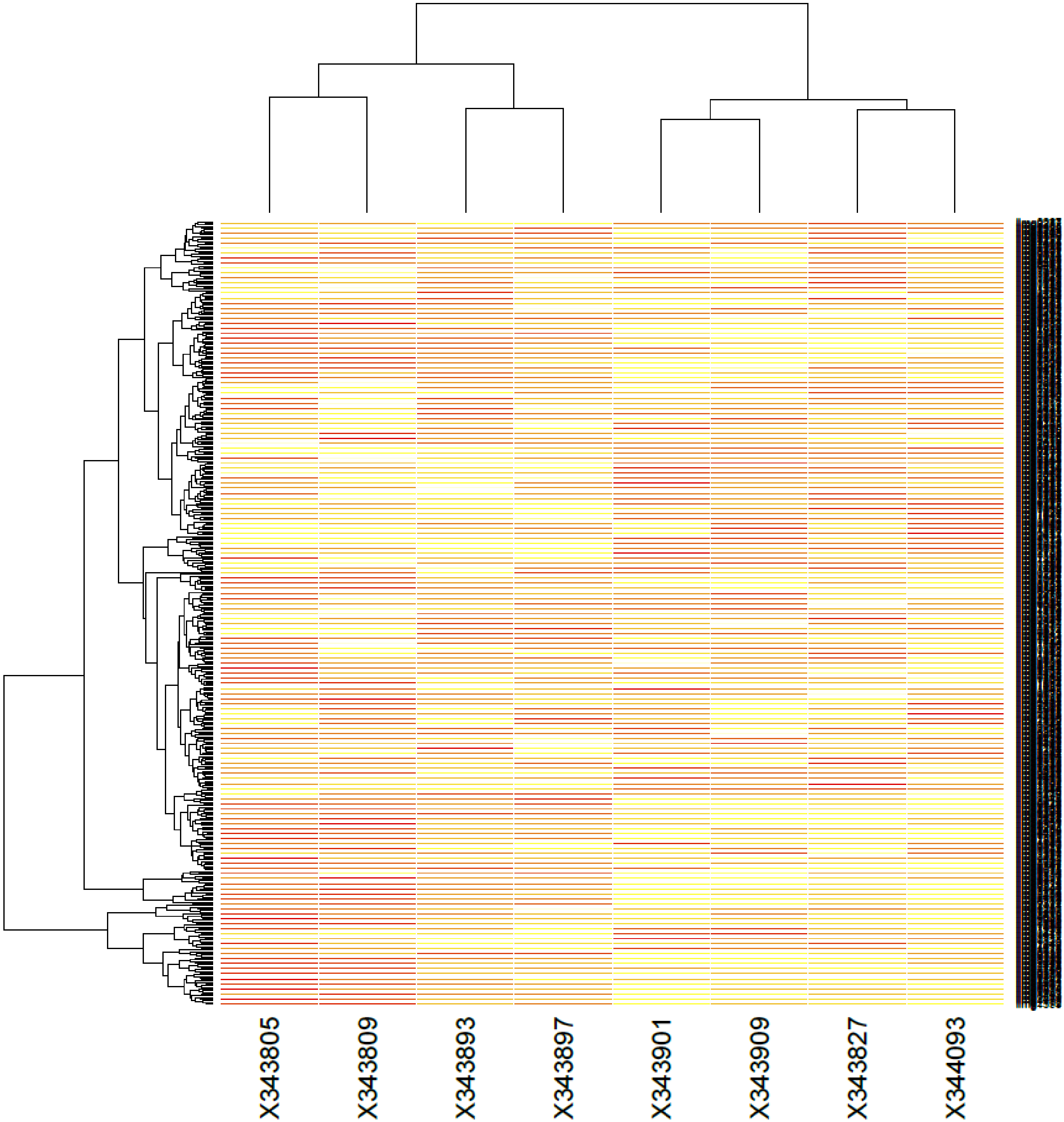

Gene expression in Regulon #15 (*ccpA*)

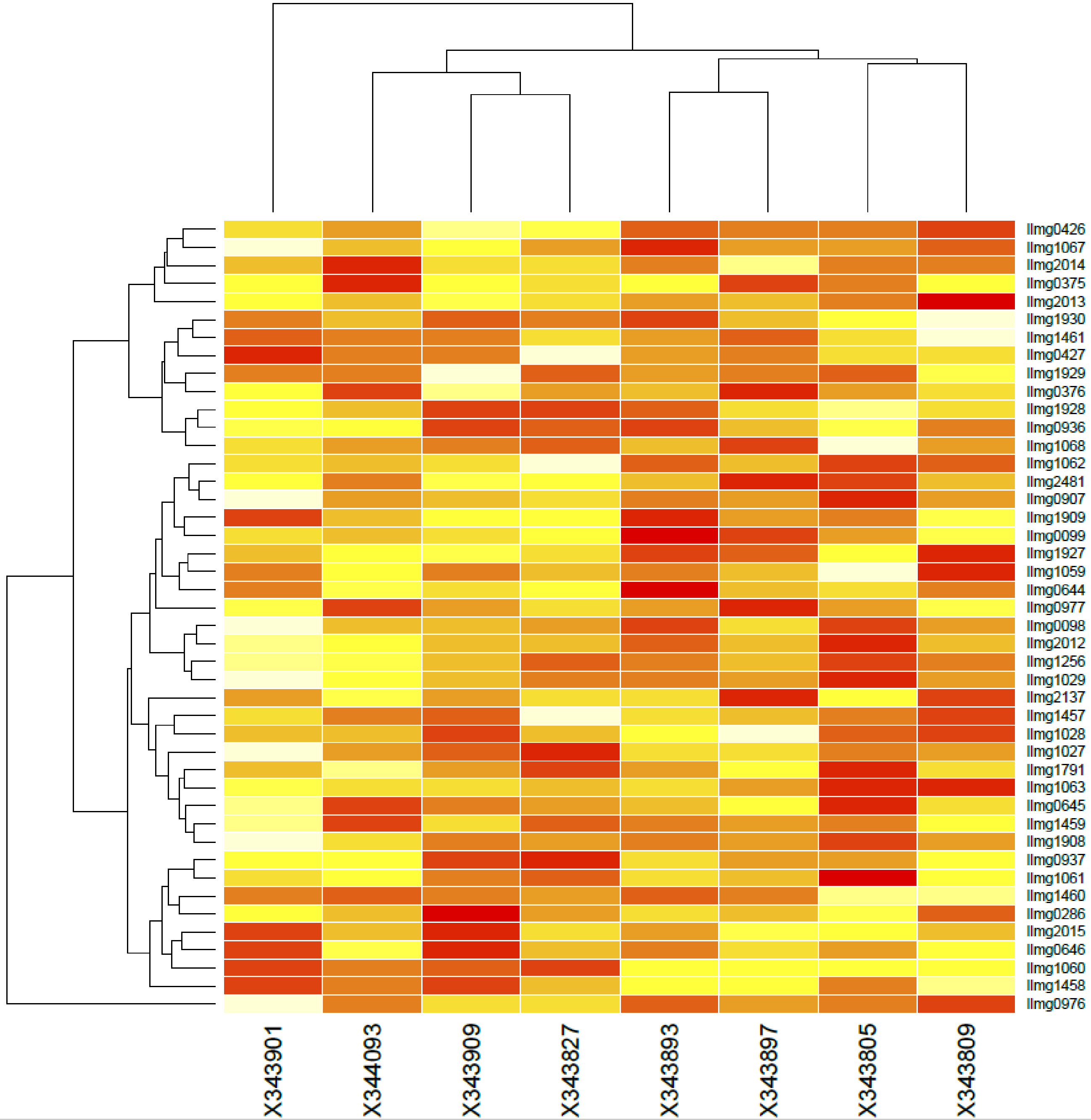

Gene expression in Regulon #31 (*hllA*)

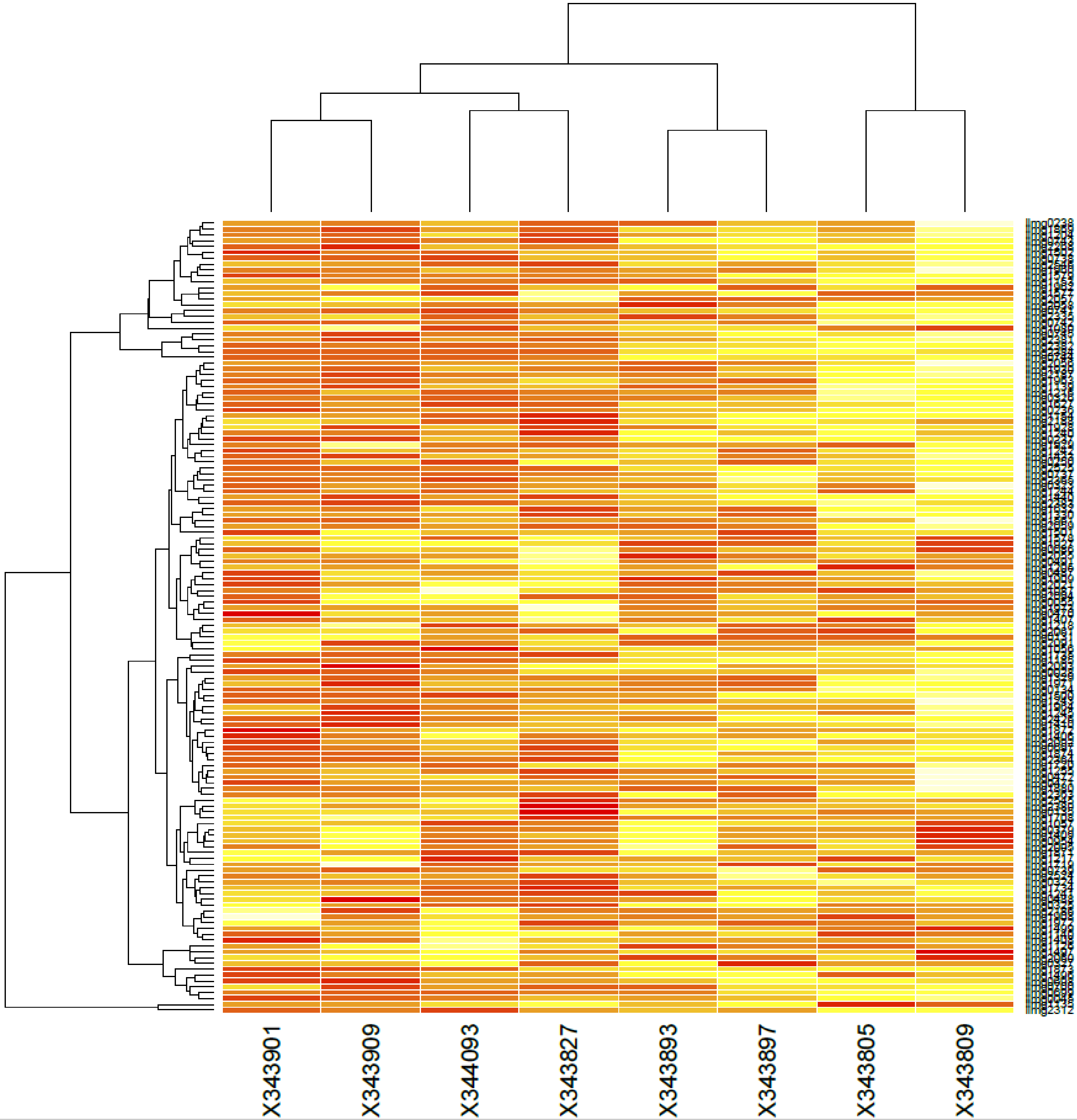

Supplement: Supplementary file 1 [file genes-09-00278-s001.zip › Figure S3 Heatmaps_for_selected_regulons.pdf]
