# Supplementary figures and images for: RECTA: Regulon Identification Based on Comparative Genomics and Transcriptomics Analysis
Source: Genes (Basel). 2018 May 30;9(6):278. doi: 10.3390/genes9060278 (PMC6027394; doi:10.3390/genes9060278)

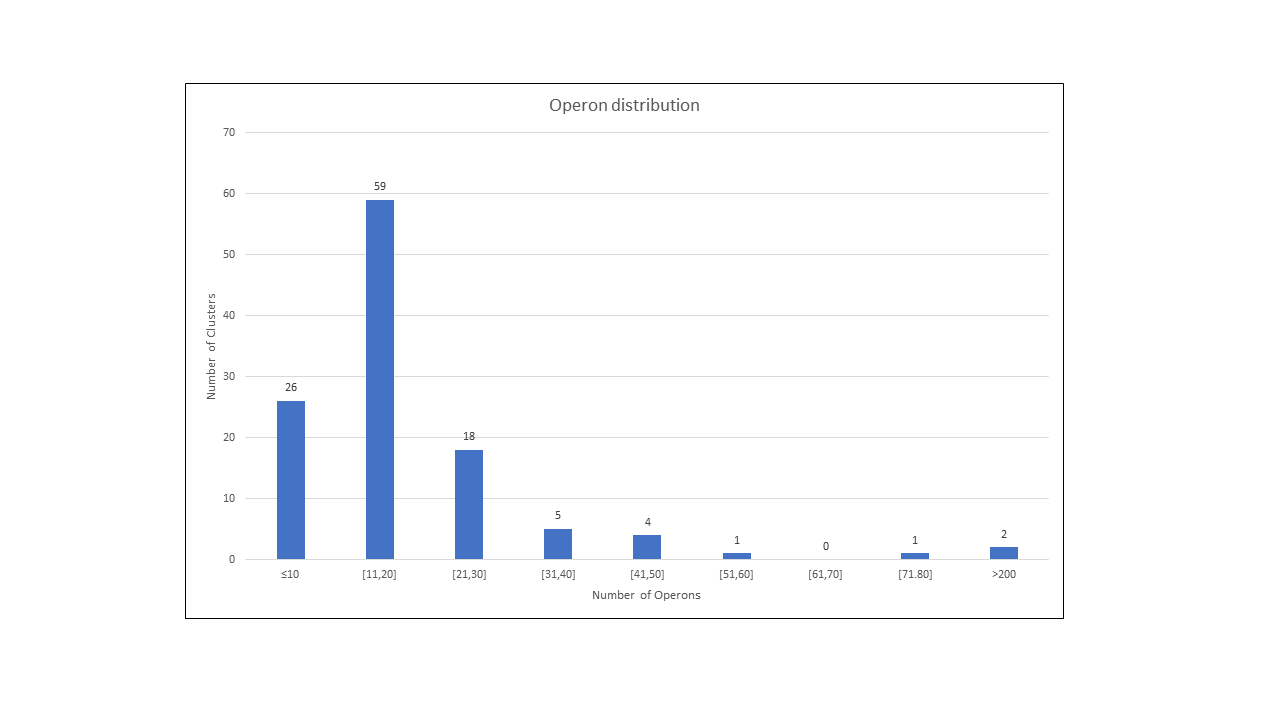

Supplement: Supplementary file 1 [file genes-09-00278-s001.zip › Figure S1 operon_distribution.tif]

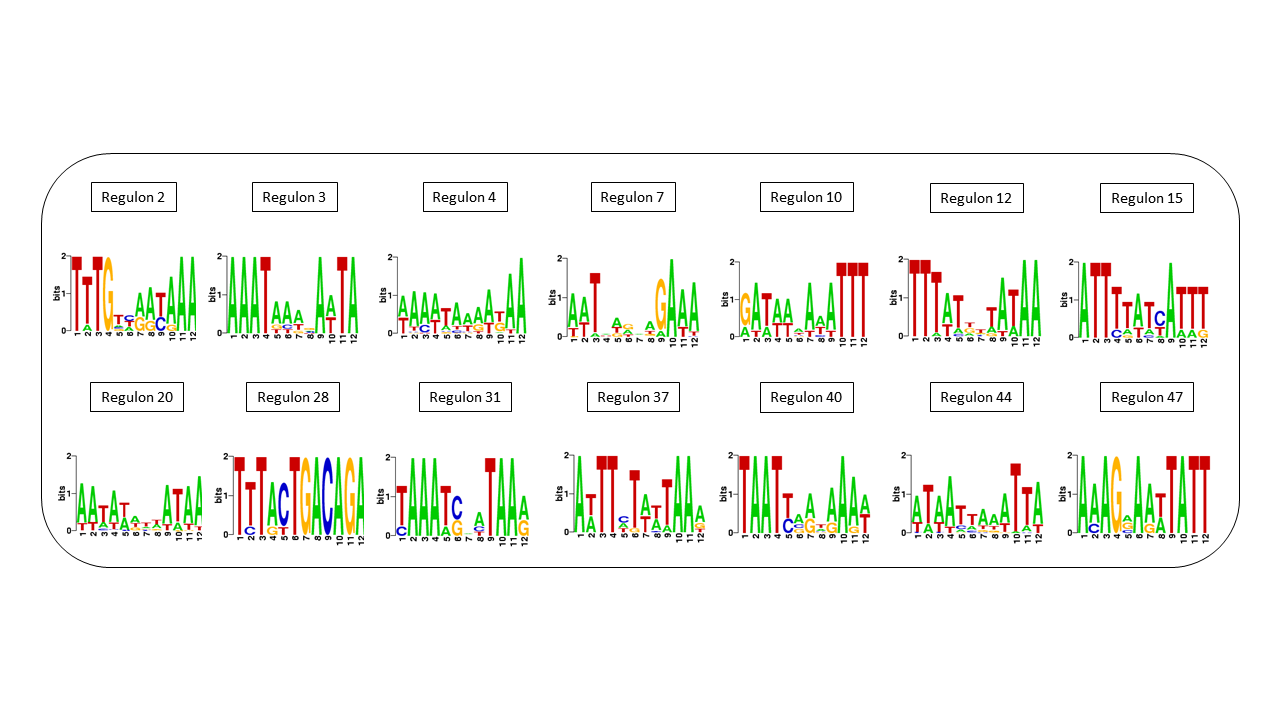

Supplement: Supplementary file 1 [file genes-09-00278-s001.zip › Figure S2 motif_logos_for_14_significant_regulons.tif]
